# Hippo Signaling: Advances in Potential Therapeutic Targets for Sinoatrial Node Disorders

**DOI:** 10.53941/ijddp.2023.100014

**Published:** 2023-12-26

**Authors:** Julianna N. Quinn, Jun Wang

**Affiliations:** 1 Department of Pediatrics, McGovern Medical School at UTHealth, The University of Texas Health Science Center at Houston, Houston, TX, 77030, USA; 2 McGovern Medical School at UTHealth, The University of Texas MD Anderson Cancer Center UTHealth Graduate School of Biomedical Sciences, The University of Texas Health Science Center at Houston, Houston, TX, 77030, USA

**Keywords:** Sinoatrial node, pacemaker, Hippo signaling, homeostasis

## Abstract

The cardiac conduction system (CCS) propagates electrical impulses, generates cardiac contractions, and ultimately ensures regular heartbeats. Disruptions within the CCS lead to cardiac arrhythmias, which are known to be the leading cause of cardiac-related mortalities in humans. The sinoatrial node (SAN) is a key component of the CCS and functions as the natural cardiac pacemaker to initiate normal cardiac impulse and conduction. The SAN is characterized by significant heterogeneity and contains various cell types, including pacemaker cells that spontaneously generate action potentials to maintain a constant beating rhythm. The fundamental Hippo signaling pathway plays a key role in heart development and regeneration. Recently, the Hippo signaling pathway is indicated as a critical pathway for maintaining SAN homeostasis, suggesting therapeutic targets for SAN disorders. This mini-review focuses on the recent molecular and mechanistic findings of Hippo’s involvement in regulating SAN homeostasis and discusses potential new therapeutic targets for SAN pathologies.

## 1. The Heterogeneous Sinoatrial Node

**Figure 1.**
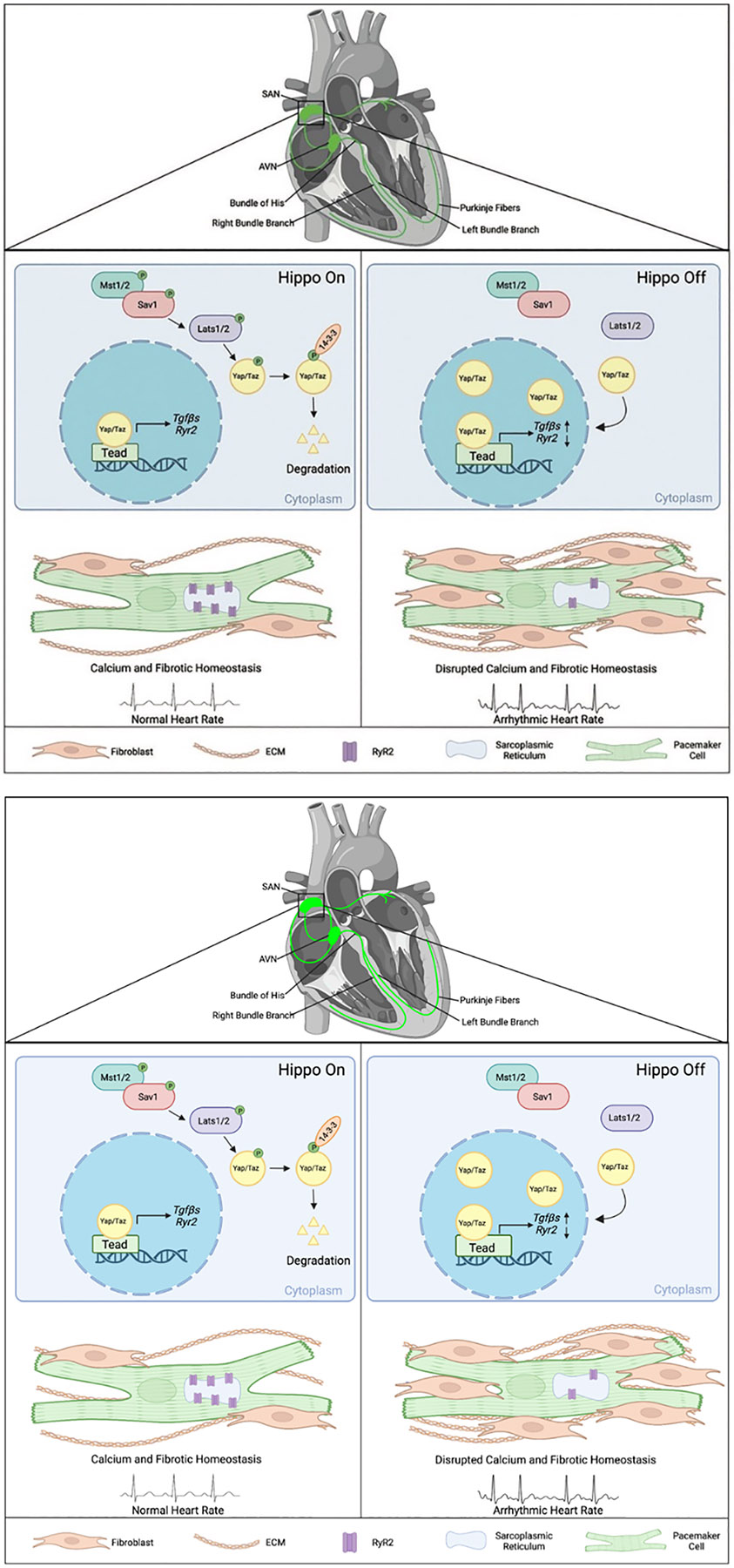
Summary of Hippo signaling-mediated mechanisms in pacemaker cells for maintaining SAN homeostasis and pathologies contributing to sinoatrial node dysfunction. The cardiac conduction system is comprised of the sinoatrial node (SAN), the atrioventricular node (AVN), the bundle of His, bundle branches, and Purkinje fibers [1]. When Hippo signaling is on, the core Hippo kinases Lats1/2 phosphorylate Yap/Taz, leading to increased 14-3-3 binding, followed by degradation in the cytoplasm [7]. Knockout of *Lats1/2* in the pacemaker cells of the SAN in adult mice results in increased nuclear Yap/Taz proteins levels, consequently causing transcriptional changes in Hippo Target genes such as *Tgfβs* and *Ryr2* [8]. Inhibition of the Hippo signaling gives rise to pathologies in the SAN, leading to cardiac arrhythmias, fibrosis, and disrupted calcium activity [8]. The figure was created using BioRender (https://biorender.com/).

The cardiac conduction system (CCS) is a tissue network of specialized muscle cells within the heart that propagates electrical impulses and orchestrates synchronized contractions of the cardiac chambers [1]. The CCS comprises distinct components: the sinoatrial node (SAN), the atrioventricular node (AVN), the bundle of His, bundle branches, and Purkinje fibers [1] (Figure 1). Pathologies affecting the CCS, known as cardiac conduction disorders, including sinoatrial node dysfunction (SND), atrioventricular block, and bundle branch block, can give rise to cardiac arrhythmias and constitute a significant cause of mortality worldwide [2]. SND (also known as sick sinus syndrome, sinus node dysfunction, or sinus node disease) causes various cardiac arrhythmias such as atrial fibrillation, cardiac arrest, and even sudden death [3, 4]. SND’s prevalence significantly increases with age and is projected to double in the next 50 years [5]. However, the pathophysiological mechanisms underlying these diseases are largely unknown due to the intricate nature of the CCS and technological limitations in studying the SAN. Lineage studies in mouse have led to the discovery of specific CCS markers and development of genetic tools to study the CCS such as the tamoxifen-inducible Hcn4CreERT2 [6], which is CCS-specific and primarily targets the pacemaker cells within the SAN.

The first generated electrical impulse originates within the heart’s natural pacemaker, known as the SAN (also known as sinus node), which is a small yet complex structure characterized by high structural and functional heterogeneity [9]. The SAN is a crescent-shaped structure that consists of a head and a tail. The head is situated at the junction of the right atria and superior vena cava, while the tail extends out near the crista terminalis [1]. This heterogeneous structure contains specialized cardiomyocytes known as pacemaker cells, a microvascular structure that varies regionally, a meshwork of interconnected cell types, including interactions with the sympathetic and parasympathetic nervous system, as well as connective components such as cardiac fibroblasts. All these components in the SAN collectively contribute to the modulation of heart rate and rhythm [9].

Pacemaker cells in the SAN are specialized cells responsible for initiating and maintaining the rhythmic contractions of the heart. These specialized cells can spontaneously generate action potentials through diastolic membrane depolarization, which then spread to the surrounding myocardium, leading to synchronized atrial contractions [4]. Pacemaker cells have distinct expression profiles of ion channels and conduction properties [10]. The generation of spontaneous action potentials and the maintenance of SAN automaticity depend on an interdependent coupled-clock system. This coupled-clock system requires interactions between two subsystem clocks: the membrane ion channels, known as the “membrane clock” (M clock), and ion channels located on the sarcoplasmic reticulum (SR), known as the Ca^2+^ clock [11]. The M and Ca^2+^ clocks collaborate through numerous interactions to drive normal pacemaker cell automaticity. During each cardiac cycle, local Ca^2+^ release from the SR occurs in response to specific signals [11]. These Ca^2+^ ions further interact with various ion channels and proteins to regulate the surface membrane potential and create an action potential [11].

The complexity and heterogeneity within the coupled-clock system has been observed in numerous studies. Distinct ion current rates, amplitudes, frequencies, and distributions have been found to regulate the electrical activity to varying extents between pacemaker cells and among cells on the SAN periphery [11-13]. Variation within SAN microvascular structure has been recently identified to play an important role in action potential firing rates. Notably, higher firing rates were observed within the superior region of the SAN, which exhibited higher vessel densities [14]. In isolated human pacemaker cells, clock-coupling could be restored in arrested pacemaker cells by stimulating β-adrenergic receptors, which are part of the sympathetic nervous system [11]. Furthermore, researchers have discovered a novel interstitial cell type expressing S100 calcium-binding protein B (S100B) within the SAN [15]. S100B is a protein known to regulate action potential firing rates and neural circuit rhythm in glial cells, and its presence has been observed to influence Ca^2+^ regulation within the SAN [15].

In addition, the fibrotic components of the SAN are significant and necessary for maintaining normal SAN homeostasis. Cardiac fibroblasts play a crucial role in providing structural and functional support to help regulate the conduction of electrical impulses by insulation [16]. However, in the context of cardiac conduction disorders, it is recognized that an increased SAN fibrosis can lead to cardiac arrhythmias and is implicated in human-related diseases such as SND [16]. Furthermore, the adult SAN responds differently to changes in ionic currents and energy metabolism, both of which are known to change with age [17,18]. Therefore, it is imperative to understand the molecular mechanisms that control adult SAN function and stability.

Transcriptional regulatory networks in the CCS were studied throughout embryonic development. The CCS are derived from shared precursor cells of atrial and ventricular working myocardium but diverge later during heart formation. Notable CCS-specific transcription factors such as Shox2 and Tbx3, along with core cardiac transcription factors during cardiac development such as Nkx2.5, Gata4/6, and Tbx5, also play essential roles in maintaining adult CCS function [19]. Signals such as Notch, AMPK, and Natriuretic Peptide Receptor signaling regulate SAN homeostasis and are implicated with SAN dysfunctions in humans [20]. Recently, the Hippo signaling pathway, recognized for its important role in cardiac development and regeneration, now has been identified as a critical pathway involved in preserving homeostasis of the SAN. This discovery has provided new insights into the molecular mechanisms through which the Hippo pathway regulates normal SAN function.

## 2. The Hippo Signaling Pathway in the Heart

The Hippo signaling pathway is an evolutionarily conserved pathway that plays essential roles in cardiac development, size, regeneration, and homeostasis [21 - 23]. The Hippo signaling is a kinase-driven cascade that includes the vital proteins: Salvador homolog 1 (Sav1) and Large tumor suppressor kinase 1/2 (Lats1/2) [7]. In the canonical Hippo-Yap pathway, the Hippo signaling functions through downstream effectors Yes-associated protein 1 (Yap) and WW-domain-containing transcription regulator 1 (Taz) [7]. When the Hippo signaling is activated, Lats1/2, the core Hippo kinases, will phosphorylate Yap/Taz, causing them to retain within the cytoplasm and later degrade due to the increase of 14-3-3 binding affinity [7]. On the other hand, when the Hippo signaling is deactivated, Yap/Taz can translocate into the nucleus and bind to the transcriptional enhanced associate domain (Tead), thereby regulating the expression of Hippo target genes [7] (Figure 1).

The canonical Hippo-Yap pathway plays crucial function in development, homeostasis, diseases, and regeneration of the heart [21 – 23]. Deleting the Hippo signaling specifically in heart results in significant cardiomegaly (enlargement of the heart) due to increased cardiomyocyte proliferation during cardiac development [24]. The adult mammalian heart has limited regeneration capacity, which hinders its ability to efficiently restore contractile function after cardiac injuries like myocardial infarction (MI) [25]. Previous studies have demonstrated that the Hippo signaling acts as a repressor for cardiomyocytes renewal and regeneration, and conversely, constitutively activation of Yap stimulates cardiomyocyte proliferation [26–33]. Deletion of Hippo signaling genes, *Salv* and *Lats1/2*, in adult mouse hearts and injured hearts induced cardiomyocyte renewal and regeneration by restoring the proliferative capacity [26]. In Salv-deficient mouse hearts, a decrease in pathological fibrosis and enhanced vasculogenesis at the scar border were observed following injury [33]. Heart function also improved following adeno-associated virus 9 (AVV9) mediated gene therapy, involving the knock down of *Salv* either at the time of injury or after ischemic heart failure [34]. Mice with conditional overexpression of *Yap5sa*, an active form of Yap with all *Lats1/2* phosphorylation sites inhibited, were found to partially reprogram the adult heart to proliferative fetal-like state. This reprogramming was achieved through alterations in the chromatin accessibility of cell-cycle genes and fetal genes [35]. Furthermore, forced overexpression of another mutated active form of Yap (S112A) in adult mouse hearts upon injury also promoted cardiomyocyte regeneration and improved cardiac function [27]. Extensive research has been conducted on the Hippo signaling pathway, revealing its critical roles in various cardiac contexts, including the myocardium [26-35] and fibroblasts [36,37], yet its role in the CCS remains largely unknown.

Chromosome abnormalities and sequence variants of the components of Hippo pathway have also been linked to cardiac defects and arrhythmias in human patients according to the patient DNA resource and database DECIPHER (Database of Genomic Variation and Phenotype in Humans using Ensembl Resources) [8]. This suggests a potential role of Hippo signaling in the CCS. However, the function of Hippo signaling in the CCS has only recently been explored.

## 3. Canonical Hippo Signaling Regulates Sinoatrial Node Homeostasis

New insights into the molecular mechanisms that maintain SAN homeostasis were recently recognized. A study conducted by Zheng et al. found that the Hippo signaling pathway is critical for adult SAN homeostasis. The core Hippo kinases *Lats1/2* were conditionally knocked out in the CCS using a tamoxifen-inducible Hcn4CreERT2 mouse [6] to assess roles of the Hippo signaling. Deletion of *Lats1/2* resulted in an increase of the active Yap protein in pacemaker nuclei in the adult SAN, indicating efficient inactivation of the *Lats1/2* [8].

*Lats1/2* mutant mice developed cardiac conduction disorders including SND presenting lower heart rates and irregular RR intervals, and atrioventricular (AV) block indicated by 24 h spontaneous ECG recording using implanted telemetry transmitters [8] (Figure 1). It was further found that calcium and fibrotic homeostasis within the SAN is regulated through the canonical Hippo signaling mediated by the downstream effectors Yap/Taz. This was confirmed through CCS specific *Yap/Taz* deletion, which rescued the phenotypes observed in *Lats1/2* mutant mice [8]. *Yap/Taz* knockout rescued the cardiac conduction disorders observed in *Lats1/2* mutant mice and reduced SAN fibrosis and fibroblast proliferation caused by *Lats1/2* deficiency [8].

The Hippo effector Yap plays a pivotal role in the regulation of various Hippo target genes. To gain insights into the specific molecular mechanisms that control SAN homeostasis, Zheng et al. utilized CUT&Tag (Cleavage Under Targets and Tagmentation) sequencing in the SANs of control and *Lats1/2* mutant mice. This genome-wide method allows for the identification of protein-DNA interactions and downstream target genes. The analysis unveiled notable differences in Yap binding peaks, and subsequent gene ontology analysis indicated that Yap likely regulates genes involved in cell-cell communication, adhesion, calcium ion activity, proliferation, and action potential [8].

## 4. Hippo Signaling is Required for Calcium Homeostasis in the Sinoatrial Node

Pacemaker cells function as specialized cardiomyocytes that generate spontaneous action potentials through distinct expression profiles of ion channels [10]. The voltage-dependent calcium ion channels are critical for maintaining and influencing pacemaker activity. As mentioned above, the coupled-clock system, which regulates SAN automaticity, consists of an M clock and a Ca^2+^ clock, each having distinct expression profiles of ion channels [11]. In pacemaker cells, the calcium ion channel ryanodine receptor 2 (RyR2), is located on the SR, a specialized endoplasmic reticulum that regulates the flow of calcium during muscle contraction [38]. Given the importance of Ca^2+^ transient rates in the proper function of pacemaker cells, and considering the aforementioned data derived from the Cut&Tag experiment showing Yap regulates calcium ion activity, further investigation revealed an impairment in calcium homeostasis within the SAN of *Lats1/2* knockout mice. This impairment resulted from decreased Ca^2+^ transient rates and reduced RyR2 levels within pacemaker cells [8]. This was validated through calcium imaging of isolated pacemaker cells during caffeine-induced treatment, revealing that the expression of RyR2 was decreased in *Lats1/2* knockout compared to the control [8]. Together, these findings suggest that the Hippo-Yap signaling pathway is likely responsible for maintaining calcium homeostasis by regulating RyR2 expression and the spontaneous firing rates of calcium (Figure 1).

## 5. Hippo Signaling Inhibition Increases Sinoatrial Node Fibrosis

As mentioned above, cardiac fibroblasts are an essential cell type within the SAN microenvironment. They provide structure and functional support as well as electrical conduction. However, under pathological conditions, CCS remodeling and excessive proliferation of fibroblasts are lead drivers of cardiac arrhythmias [16].

Cardiac fibroblasts play a crucial role in maintaining the cardiac structural integrity by disposing of extracellular matrix (ECM) proteins that provide scaffolds for both functional and structural support throughout the heart [39]. Among these proteins, collagens are particularly important, but excess amounts can lead to abnormalities. Within the heart, two dynamic forms of cardiac fibrosis exist: reparative and reactive. Reparative fibrosis emerges as a response to cardiomyocyte loss, triggering the proliferation of the surrounding fibroblasts [39]. Reactive fibrosis, on the other hand, occurs when mediators from the myocardium stimulate fibroblasts to deposit the ECM proteins [39]. Canonical Hippo signaling has been implicated in cardiac fibroblast development and differentiation from embryonic epicardial precursors. Deletion of *Lats1/2* in the epicardium Cre driver Wt1CreERT resulted in abnormal Yap activation, disrupting the transition from epicardial to mature cardiac fibroblasts [37]. Hippo signaling also plays a critical role in adult cardiac fibroblast homeostasis. Conditional deletion of *Lats1/2* in mature cardiac fibroblasts induced proliferation and led to cardiac dysfunctions [36].

In the context of the SAN, Hippo signaling has been demonstrated to play a critical role in fibroblast regulation upon *Lats1/2* deletion in pacemaker cells. Fibrosis-regulation genes, such as Collagen 1, Vimentin, Smooth Muscle Actin, and Periostin, were found to be upregulated at both mRNA and protein levels in the SANs of *Lats1/2* knockout mutants compared to controls [8]. The Cut&Tag experiment further revealed paracrine fibrosis inducers *Tgfβ1* and *Tgfβ3* (transforming growth factor-β), as specific Yap-Tead targets and potential modulators for SAN fibrosis [8]. The loss of *Lats1/2* promoted fibroblast remodeling and proliferation within the SAN [8], substantiated by an increase in proliferative vimentin-positive fibroblasts and the accumulation of the ECM protein Collagen 1 [8]. This increase in SAN fibrosis has the potential to disrupt proper pacemaker function by impairing conduction and electrical activity, potentially contributing to the observed cardiac dysfunctions (Figure 1).

## 6. New Perspectives for Therapeutics Targeting Sinoatrial Node-related Diseases

To further understand potential mechanisms of SAN fibrosis, previously discussed CUT&Tag experiment revealed that paracrine fibrosis inducers *Tgfβ1* and *Tgfβ3* were upregulated due to increased Yap binding activities in *Lats1/2* knockout mice [8]. Furthermore, *Lats1/2* knockout mice exhibited significantly increased expression of *Tgfβ1* and pSmad3 (indicator for Tgfβ pathway activity), suggesting that the increased active Yap upon *Lats1/2* knockout in the SAN functions as a Tgfβ signaling activator, mediating fibroblast proliferation and remodeling [8]. Tgfβ is known to play diverse roles in cardiac diseases, including cardiac abnormalities, hypertrophy, remodeling, and fibrosis [40]. Upon treatment with the Tgfβ1 receptor inhibitor: SB431542, *Lats1/2* knockout mice had partially rescued fibrosis. This finding suggests that *Lats1/2* deficiency induces SAN fibrosis through activation of the Tgfβ signaling pathway (Figure 1). The Tgfβ pathway activity has been extensively studied as a shared mediator for cancer progression and a promoter for fibrotic conditions in multiple organs, including the heart [41]. Targeting this pathway has been considered for antifibrotic therapies, and recent developments have centered on small molecule inhibitors for treating cardiac fibrosis [41]. Understanding the role of Tgfβ pathway and the confirmed efficacy of treatment using a Tgfβ1 receptor inhibitor offers new and promising treatment strategies for treating SAN fibrosis.

Maintaining homeostasis in the adult SAN is crucial for the proper function of pacemaker cells. Several intracellular signals such as Notch, AMPK, Natriuretic Peptide Receptor [20], and now Hippo signaling [8], have been identified as essential for maintaining the function of the CCS. Disruptions in these pathways have been linked to pathologies consistent with SAN dysfunction in humans [20]. Given the correlation of the Hippo pathway with cardiac regeneration, development [26–35], fibrosis [36,37], and its role as a regulator of SAN homeostasis [8], the Hippo pathway has garnered significant attention as a potential therapeutic target. Recent efforts have focused on activation of Yap proteins or inhibition of upstream Hippo signaling using various strategies. Drug development has also aimed to inhibit the core Hippo kinases; however, it should be noted that these kinases have additional roles in various cell signaling pathways and may lead to adverse effects [42,43].

The Hippo pathway was studied extensively within the context of cardiac regeneration following injury. Inhibition of the Hippo signaling by manipulation of core Hippo proteins has emerged as a promising target for promoting cardiomyocyte renewal. A study by Liu et al. used adeno-associated virus 9 (AVV9) mediated gene therapy to knock down *Sav1* in cardiomyocytes post-MI in pigs [33]. The viral vector, AAV9-*Sav1*-short hairpin RNA (shRNA), was administered two weeks after the injury and was found to alleviate abnormal systolic function by increasing cardiomyocyte renewal [33]. Notably, inhibition of the Hippo signaling upon cardiac injury promoted cardiomyocyte proliferation, aided in the formation of new cardiac blood vessels, and reduced pathological fibrosis [33]. MI often induces cardiac arrhythmias, such as refractory ventricular arrhythmias, which are prevalent in MI patients and a leading cause of heart failure and mortality [32]. In a later study by Zhang et al. utilizing the same viral vector, AAV9-*Sav1*-shRNA, it was demonstrated that inhibition of the Hippo signaling attenuated MI-related cardiac arrhythmias, specifically refractory arrhythmias [32].

## 7. Conclusion

The components of the CCS play a crucial role in generating electrical impulses and synchronized contractions of the cardiac chambers, which ultimately creating your heartbeat. Maintaining homeostasis within this system is vital to prevent fatal cardiac dysfunctions including SND. Various factors, such as metabolism and ionic currents within the adult SAN, are recognized to change with age [17, 18], potentially disrupting normal SAN automaticity and leading to heart failure. Understanding the cellular and molecular mechanisms that underlie pathologies related to SND can advance our understanding of the complex signaling within the CCS and offer potential therapeutic targets with minimal adverse effects.

The Hippo pathway, known to be linked with human patients with cardiac defects and already an active target for therapies in the context of MI, now holds promise as a potential target for CCS therapies, particularly in studies related to aging. Additionally, the regulation of Tgfβ activity by Hippo pathway suggested other novel treatment strategies by targeting downstream signaling pathways modulated by Hippo. Further investigation is needed to understand the cellular mechanisms responsible for maintaining homeostasis in other CCS components, such as the atrioventricular node (AVN) and different CCS cell types. Moreover, age-related studies that focus on Hippo’s involvement in the SAN and therapies specifically targeting Hippo in the SAN require further evaluation in aged mice and other model systems to pave the way for future studies.
